# Racial Disparities in Patients With COVID-19 Infection: A National Inpatient Sample Analysis

**DOI:** 10.7759/cureus.35039

**Published:** 2023-02-15

**Authors:** Ufuk Vardar, Ayodeji Ilelaboye, Mukunthan Murthi, Ramtej Atluri, Dae Yong Park, Parnia Khamooshi, Pius E Ojemolon, Hafeez Shaka

**Affiliations:** 1 Internal Medicine, John H. Stroger, Jr. Hospital of Cook County, Chicago, USA; 2 Internal Medicine, Ladoke Akintola University of Technology, Ogbomosho, NGA

**Keywords:** length of hospital stay (los), national inpatient sample database, inpatient mortality, racial and ethnic disparities, covid 19

## Abstract

Introduction

Evidence suggests the COVID-19 (coronavirus disease 2019) pandemic highlighted well-known healthcare disparities. This study investigated racial disparities in patients with COVID-19-related hospitalizations utilizing the US (United States) National Inpatient Sample (NIS).

Methodology

This was a retrospective study conducted utilizing the NIS 2020 database. The NIS was searched for hospitalization of adult patients with COVID-19 infection as a principal diagnosis using ICD-10 (International Classification of Diseases, Tenth Revision) codes. We divided the NIS into four major racial/ethnic groups: White, Black, Hispanic, and others. The primary outcome was inpatient mortality, and the secondary outcomes were the mean length of stay, mean total hospital charges, development of sepsis, septic shock, use of vasopressors, acute respiratory failure, acute respiratory distress syndrome, acute kidney failure, acute myocardial infarction, cardiac arrest, deep vein thrombosis, pulmonary embolism, cerebrovascular accident, and need for mechanical ventilation.

Results

Compared to White patients, Hispanic patients had higher adjusted inpatient mortality odds (aOR [adjusted odds ratio]: 1.25, 95% CI 1.19-1.33, p<0.001); however, Black patients had similar adjusted mortality odds (aOR: 0.96, 95% CI 0.91-1.01, p=0.212). Black patients and Hispanic patients had a higher mean length of stay (8.01 vs 7.13 days, p<0.001 and 7.67 vs 7.13 days, p<0.001, respectively), adjusted odds of cardiac arrest (aOR: 1.53, 95% CI 1.37-1.71, p<0.001 and aOR: 1.73, 95% CI 1.54-1.94, p<0.001), septic shock (aOR: 1.23, 95% CI 1.13-1.33, p<0.001 and aOR: 1.88, 95% CI 1.73-2.04, p<0.001), and vasopressor use (aOR: 1.32, 95% CI 1.14 - 1.53, p<0.001 and aOR: 1.87, 95% CI 1.62 - 2.16, p<0.001).

Conclusion

Our study showed that Black and Hispanic patients are at higher risk of adverse outcomes compared to White patients admitted with COVID-19 infection.

## Introduction

The initial outbreak of severe acute respiratory syndrome coronavirus 2 (SARS-CoV-2) infection occurred in December 2019. Since then, SARS-CoV-2 has spread rapidly and caused a global pandemic [1]. It continues to impact the health, economy, and quality of life of individuals and societies globally. By October 12, 2022, there were 96,581,755 confirmed cases and 1,057,975 deaths in the United States alone [2].

Health disparities, defined as a higher burden of illness, injury, disability, or mortality among one group relative to another, are well known and documented in the United States [3]. The COVID-19 pandemic highlighted the prominent impact of social determinants of health [4]. The health effects of COVID-19 have been unevenly distributed across the United States [5]. Racial/ethnic disparities are observed in healthcare utilization and outcomes of populations during the COVID-19 pandemic, and evidence has emerged that the pandemic is disproportionately affecting people from Black, Hispanic, and minority ethnic communities [6,7].

The primary objective of this study was to identify how COVID-19 affected major racial/ethnic groups in the United States on a national level utilizing the National Inpatient Sample (NIS) database for 2020.

## Materials and methods

Design and data source

This was a retrospective cohort study, using the NIS database for 2020. The NIS is the largest database of hospital inpatient stays in the United States derived from billing data submitted by hospitals to statewide data organizations across the United States, covering 98% of the US population. It contains discharge data from a 20% stratified sample of community hospitals and is a part of the Healthcare Quality and Utilization Project (HCUP), sponsored by the Agency for Healthcare Research and Quality. The International Classification of Diseases, Tenth Revision, Clinical Modification/Procedure Coding System (ICD-10-CM/PCS) was used in the coding. Diagnoses are divided into principal diagnosis and secondary diagnosis. A principal diagnosis is the main ICD-10 code for hospitalization. Secondary diagnoses are any ICD-10 code other than the principal diagnosis billed for that hospitalization.

Study population

We queried the NIS 2020 database for patients who had a principal discharge diagnosis of COVID-19 infection. Patients who were younger than 18 years of age or who had elective admissions were excluded from our study. The ICD-10-CM codes used for COVID-19-related hospitalizations were U.071, U.00, U.49, U.50, U.85, J.1282, and B.342. Per coding guidelines, these codes were based on documentation by the provider or documentation of a positive COVID-19 test result. The ICD-10-CM diagnosis codes for COVID-19 were implemented beginning April 1, 2020 [8]. Coding for race in NIS combines “race” and “ethnicity” provided by the data source into one data element (RACE). If both “race” and “ethnicity” were available, ethnicity was preferred over race in setting the HCUP value for “RACE". For this analysis, race/ethnicity was classified as White, Black, Hispanic, and others (Asian or Pacific Islander, Native American, others) as employed in prior NIS-based publications [9-11].

Outcome measures

The primary outcome of our study was comparing inpatient mortality among race groups. Secondary outcomes included mean length of stay, mean total hospital charges, development of sepsis, septic shock, use of vasopressors, acute respiratory failure, acute respiratory distress syndrome (ARDS), acute kidney failure, acute myocardial infarction, cardiac arrest, deep vein thrombosis, pulmonary embolism, cerebrovascular accident, and need for mechanical ventilation.

Statistical analysis

The data were analyzed using Stata® Version 14 software (StataCorp, Texas, USA). Comorbidities were calculated as proportions of the cohorts and the chi-square test was used to compare these characteristics among different racial groups. The Deyo modification of the Charlson Comorbidity Index (CCI) was used to identify the burden of comorbid diseases [12]. CCI, as a summary comorbidity measure, was used as a potential confounder in the multivariate regression analysis, given comparable performance to each component of CCI used separately [13,14]. Variables obtained from the literature search (age, sex, race/ethnicity, median yearly income in the patient’s zip code, hospital location [rural or urban], geographic region [Northeast, Midwest, West, or South], hospital teaching status, and hospital bed size, smoking history, obesity, malnutrition, anemia, and pulmonary hypertension) were tested with a univariate screen. Variables with p-values <0.01 were subsequently included in the final multivariate regression model (stricter entry criteria were implemented given large database analysis). This was done to avoid overpowering and avoid variables attaining statistical significance while only marginally changing the outcome [15].

Using predictive margins analysis in our multivariate regression model, we obtained adjusted mortality rates, mean total hospital charges, mean length of stay, and the rates of the other secondary outcomes. We present both the crude and adjusted values for these outcomes in this study.

Ethical considerations

The NIS is a retrospective database that protects patient confidentiality, lacking individual or hospital identifiers. This study was therefore exempt from our Institutional Review Board approval.

## Results

Patient characteristics

NIS database for 2020 contained over 32 million weighted hospital discharges, of which 1,019,325 were adults (persons 18 years and above), non-elective admissions, and had a principal diagnosis of COVID-19 infection, which satisfied the inclusion criteria.

Out of all patients admitted with COVID-19 infection, White patients comprised 52.4% and were significantly older compared to Black patients and Hispanic patients (68.9 years vs 61.2 years vs 58.1 years, p<0.001). Black patients had a higher proportion of females compared to White patients and Hispanic patients (53.3% vs 46.9% vs 43.6%). Black patients had the highest proportion of diabetes mellitus, hypertension, obesity, and malignancy, while White patients had the highest proportion of history of smoking, chronic obstructive pulmonary disease, and dependency on oxygen. Baseline patient and hospital characteristics are shown in Table 1.

**Table 1:** National Demographic and Clinical Characteristics of COVID-19 Hospitalizations in 2020

Variable	White Patients	Black Patients	Hispanic Patients	Others	p-Value
COVID-19 infection (N=1,019,325)	n=534,126	n=189,575	n=210,980	n=84,644	
Mean age (years)	68.9	61.2	58.1	61.3	<0.001
Female (%)	46.9	53.3	43.6	44.8	<0.001
Insurance type (%)					<0.001
Medicare	65.7	51.4	38.5	42.7	
Medicaid	5.4	16.4	26.3	19.6	
Private	27.1	30.9	32.4	35.3	
Uninsured	1.8	1.3	2.8	2.4	
Charlson Comorbidity Index Score (%)					<0.001
0	25.5	23.1	35.3	31.8	
1	26.5	26.2	31.2	31.3	
2	17.7	16.5	13.6	14.1	
≥3	30.3	34.2	19.9	22.8	
Median household income (%)					<0.001
< $49,999	27.1	51.9	37.5	26.2	
≥ $50,000 to < $64,999	29.8	22.7	27.3	23.9	
≥ $65,000 to < $85,999	24.1	15.5	22.9	24.3	
≥ $86,000	19.0	9.9	12.3	25.6	
Hospital region (%)					<0.001
Northeast	17.2	18.1	17.7	19.2	
Midwest	29.9	19.9	9.8	17.3	
South	40.3	57.1	37.6	27.8	
West	12.6	4.9	34.9	35.7	
Hospital bed size (%)					<0.001
Small	26.3	24.7	22.9	22.2	
Medium	28.9	27.8	30.5	29.7	
Large	44.8	47.5	46.6	48.1	
Location/teaching status of hospital (%)					<0.001
Rural	15.7	8.1	3.6	5.0	
Urban non-teaching	21.0	14.4	20.8	19.3	
Urban teaching	63.3	77.5	75.6	75.7	
Comorbidities (%)					
Diabetes mellitus	36.0	47.9	45.0	40.8	<0.001
Hypertension	70.2	76.8	56.0	61.5	<0.001
Smoking	27.0	18.2	13.7	14.6	<0.001
Congestive heart failure	19.5	19.6	9.7	11.3	<0.001
Chronic kidney disease	20.6	27.1	14.5	16.8	<0.001
Obesity	25.8	33.6	29.2	27.6	<0.001
Pulmonary hypertension	2.8	3.0	1.5	1.8	<0.001
History of cerebrovascular accident	3.9	3.5	2.7	3.8	0.072
Chronic obstructive pulmonary disease	17.5	11.4	4.9	6.7	<0.001
Dependency on oxygen	4.9	3.1	2.3	2.5	<0.001
Liver disease	3.5	3.1	5.5	5.0	<0.001
Anemia	18.3	26.6	18.8	19.5	<0.001
Malignancy	4.2	3.3	2.1	2.5	<0.001

Primary outcome: inpatient mortality

The crude inpatient mortality rate of COVID-19 infection for the total cohort was 11.10%. White patients had the highest crude mortality rate (11.63%); however, when the mortality rate was adjusted for other variables using predictive margins analysis in our logistic regression model, Hispanic patients had the highest adjusted mortality rate (10.41%). When compared to White patients (taken as reference), the adjusted odds ratio for mortality was 0.96 (95% CI 0.91-1.01, p=0.212) for Black patients, 1.25 (95% CI 1.19-1.33, p<0.001) for Hispanic patients, and 1.22 (95% CI 1.14-1.31, p<0.001) for other races.

Secondary outcomes

The total length of hospitalization and the total hospital charges between the Black patient and the Hispanic patient groups were compared to White patients using a multivariate linear regression model. Compared to White patients, Black patients and Hispanic patients had a higher mean length of stay (8.01 vs 7.13 days, p<0.001, and 7.67 vs 7.13 days, p<0.001, respectively). Hispanic patients had significantly higher mean total hospital charges compared to White patients ($104,826 vs $67,682, p<0.001). However, there was no difference in mean total hospital charges between Black patients and White patients. Among different racial/ethnic groups, the remaining secondary outcomes are demonstrated in Table 2, and adjusted odds ratios are visualized in Table 3.

**Table 2:** National Clinical Outcomes for COVID-Related Hospitalizations Stratified by Race

Outcome	Crude Outcomes				Adjusted Outcomes			
	White Patients	Black Patients	Hispanic Patients	Others	White Patients	Black Patients	Hispanic Patients	Others
Primary outcome								
Inpatient mortality (%)	11.63	9.86	10.51	11.60	8.46	8.22	10.41	10.16
Secondary outcomes								
Length of stay, mean (days)	7.13	7.67	8.01	7.87	6.73	7.06	7.83	7.54
Total hospital charges, mean US$	67,682	74,958	104,826	97,439	72,086	74,860	101,205	91,968
Sepsis (%)	5.2	6.7	8.1	8.2	5.1	5.9	7.8	7.6
Septic shock (%)	2.6	3.5	4.6	4.5	2.4	3.0	4.5	4.2
Acute respiratory failure (%)	59.1	51.5	59.8	60.3	57.7	50.8	57.5	57.9
Acute respiratory distress syndrome (%)	4.7	4.6	6.6	5.2	4.7	4.5	6.6	6.3
Cardiac arrest (%)	1.6	2.7	2.6	2.4	1.4	2.2	2.5	2.3
Acute kidney failure (%)	24.6	35.3	19.5	22.7	19.8	29.8	20.2	21.3
Deep vein thrombosis (%)	1.9	2.2	1.8	1.7	1.7	2.1	1.7	1.5
Pulmonary embolism (%)	2.9	3.4	2.1	2.2	2.8	3.4	2.0	2.1
Cerebrovascular accident (%)	0.6	0.9	0.7	1.0	0.6	0.8	0.9	0.11
Acute myocardial infarction (%)	3.5	3.4	3.0	3.6	3.1	3.0	3.7	4.0
Need for mechanical ventilation (%)	6.7	5.6	6.2	6.5	6.2	5.2	6.2	6.4
Use of vasopressors (%)	1.4	1.9	2.5	2.4	1.3	1.8	2.5	2.1

**Table 3:** Adjusted Odds Ratio of National Clinical Outcomes of Black Patients, Hispanic Patients, and Others Compared to White Patients in COVID-Related Hospitalizations *Signifies adjusted mean difference. aOR, adjusted odds ratio.

Outcome	Black Patients		Hispanic Patients		Others	
	aOR (95% CI)	p-Value	aOR (95% CI)	p-Value	aOR (95% CI)	p-Value
Primary outcome						
Inpatient mortality	0.96 (0.91-1.01)	0.212	1.28 (1.21-1.36)	<0.001	1.25 (1.16-1.34)	<0.001
Secondary outcomes						
Length of stay, mean, days	+0.33* (0.21-0.45)	<0.001	+1.09* (0.92-1.25)	<0.001	+0.80* (0.61-0.99)	<0.001
Total hospital charges, mean, US$	+2773* (-56 - +5602)	0.055	+29,118* (24,730-33,505)	<0.001	+19,881* (14,078-25,684)	<0.001
Sepsis (%)	1.19 (1.11-1.27)	<0.001	1.59 (149-1.70)	<0.001	1.56 (1.45-1.68)	<0.001
Septic shock (%)	1.23 (1.13-1.33)	<0.001	1.88 (1.73-2.04)	<0.001	1.73 (1.57-1.90)	<0.001
Acute respiratory failure (%)	1.20 (1.02-1.41)	0.024	1.13 (0.95-1.35)	0.136	1.11 (0.91-1.35)	0.294
Acute respiratory distress syndrome (%)	0.94 (0.87-1.02)	0.163	1.41 (1.30-1.54)	<0.001	1.36 (1.23-1.49)	<0.001
Cardiac arrest (%)	1.53 (1.37-1.71)	<0.001	1.73 (1.54-1.94)	<0.001	1.57 (1.37-1.79)	<0.001
Acute kidney injury (%)	1.94 (1.87-2.01)	<0.001	1.02 (0.98-1.06)	0.189	1.11 (1.06-1.16)	<0.001
Deep vein thrombosis (%)	1.22 (1.11-1.35)	<0.001	1.02 (0.90-1.16)	0.679	0.90 (0.78-1.03)	0.131
Pulmonary embolism (%)	1.24 (1.15-1.34)	<0.001	0.71 (0.65-0.78)	<0.001	0.72 (0.64-0.81)	<0.001
Cerebrovascular accident (%)	1.25 (1.08-1.46)	0.003	1.27 (1.08-1.48)	0.002	1.75 (1.44-2.13)	<0.001
Acute myocardial infarction (%)	0.94 (0.86-1.03)	0.219	1.18 (1.05-1.32)	0.004	1.30 (1.16-1.32)	<0.001
Need for mechanical ventilation (%)	0.83 (0.76-0.90)	<0.001	1.00 (0.92-1.09)	0.874	1.04 (0.94-1.16)	0.393
Use of vasopressors (%)	1.32 (1.14-1.53)	<0.001	1.87 (1.62-2.16)	<0.001	1.59 (1.37-1.86)	<0.001

## Discussion

According to our study, the proportion of White patients admitted for COVID-19 was significantly less than the adult White population of the US represented in the whole NIS (52.4% vs 63.6%, p<0.001). Strikingly, the proportions of Black patients and Hispanic patients with COVID-19-related hospitalizations were significantly higher compared to their proportions in the whole NIS (18.6% vs 15.8%, p<0.001, and 20.6% vs 13.1%, p<0.001). This signifies that a lower proportion of White patients and a higher proportion of Black patients and Hispanic patients were hospitalized for COVID-19 compared to the entire US hospitalizations. This finding was comparable to the latest available CDC data, which suggest that race and ethnicity are risk markers for worse outcomes for COVID-19 patients [16]. The above finding is likely tied to a complex mechanism driving different healthcare outcomes of different races, connections, and consequences of socioeconomic disparities, including the type of work, location, and access to health care [17,18]. A study conducted by Abedi et al. [19] suggests that counties in the United States with higher and more diverse populations have a higher rate of SARS-CoV-2 infection, whereas counties with grocery mobility (lack of food deserts) and work mobility are associated with lower rates of infection. Another study by Dey et al. [20] hypothesized that air pollution might increase vulnerability to COVID-19 resulting in hospitalization and/or death, which may be linked to racial disparities since zip codes with high levels of fine particulate matter (PM2.5) mostly predominantly consisted of racial/ethnic minorities.

Crude mortality and adjusted mortality were significantly different among racial/ethnic groups in patients with COVID-19-related hospitalizations. This discrepancy between the two approaches is likely secondary to other confounders affecting mortality, which were also significantly different among our cohort groups. For example, White patients were significantly older than the other groups and older age is associated with higher mortality. These findings are comparable to prior studies, such as findings from a cross-sectional study by Acosta et al. [21] involving 143,342 patients which demonstrated significant differences in inpatient mortality among different racial groups, and Hispanic patients and Black patients had a higher risk of inpatient mortality; however, a study conducted by Yehia et al. [22], with 92 US hospitals involved, suggested that there was no mortality difference between Black patients and White patients (HR 0.93, 95% CI 0.80-1.09).

We found that Black patients and Hispanic patients had a significantly higher length of stay and increased odds of sepsis, septic shock, and use of vasopressors compared to White patients. Hispanic patients had higher mean total hospital charges and increased odds of developing ARDS compared to White patients, a well-known complication of COVID-19 infection. Our findings build on previous COVID-NET studies, and other assessments that found that Black and Hispanic populations are disproportionately affected by COVID-19-related hospitalizations [23,24].

Our study has several strengths. The data were sourced from a large national database, providing a large sample size that enabled us to compare mortality outcomes. The nature of the database allows us to provide insights into the comparison of baseline demographics and hospital outcomes between different racial/ethnic groups to statistically significant levels.

There are some limitations to our study. First, the database we utilized has limitations related to coding, missing data, and dependency on inpatient admissions (does not involve outpatient COVID-19 infections), among others. NIS database studies involve hospitalizations and not individual patients, so patients admitted multiple times will be counted separately. Second, COVID-19 treatment evolved significantly over the past years. This database includes patients from 2020; hence, treatments involved in our cohort could be outdated compared to current guidelines and management of COVID-19 infections. Previous studies conducted in 2020 and 2021 reveal similar disparities in racial/ethnic populations regarding COVID-19 outcomes, and this may suggest that the newly developed therapies may also be poorly distributed among different groups, tied closely to availability and access to health care [25,26].

## Conclusions

In summary, among adults excluding elective admissions, Black, Hispanic, and other non-White ethnic/racial groups are at a higher risk of adverse outcomes including inpatient mortality, respiratory complications, and length of hospital stay when compared to White patients in patients with COVID-19-related hospitalizations. There were significant disparities in outcomes across various sociodemographic groups. This discrepancy identified warrants further randomized studies into possible unadjusted confounders to address healthcare disparities. Our findings may help identify potential gaps in health care involving COVID-19 patients.

## Appendices

**Table 4:** List of the ICD-10-CM and ICD-10-PCS codes used

Diagnosis	ICD-10 codes
Diabetes mellitus	E08, E10, E11, E13
Hypertension	I10
Smoking	F17, T65, Z72.0, O99.33, Z87.891
Congestive heart failure	I09.81, I11.0, I13.0, I31.2, I50
Chronic kidney disease	N18
Obesity	E66
Pulmonary hypertension	I27.0, I27.2
History of cerebrovascular accident	I69, Z86.73
Chronic obstructive pulmonary disease	J41, J42, J43, J44
Dependency on oxygen	Z9981
Liver disease	K70.2, K70.3, K71.7, K74, K76.1, P78.81, E83.110
Anemia	D50, D51, D52, D53
Malignancy	Z85
Sepsis	A40.x, A41.x, R652.x
Septic shock	R6521
Acute respiratory failure	J960.x, J962.x, J969.x
Acute respiratory distress syndrome	J80
Cardiac arrest	I46.x
Acute kidney injury	N17.x
Deep vein thrombosis	I824.x, I825.x, I826.x, I827.x
Pulmonary embolism	I26
Cerebrovascular accident	I63.x
Acute myocardial infarction	I21.x, I22.x
Need for mechanical ventilation	5A1935Z, 5A1945Z, 5A1955Z
Use of vasopressors	3E030XZ, 3E033XZ, 3E040XZ, 3E043XZ, 3E050XZ, 3E053XZ, 3E060XZ, 3E063XZ
